# Supplementary material for: The Thermosynechococcus Genus: Wide Environmental Distribution, but a Highly Conserved Genomic Core
Source: Microbes Environ. 2021 May 1;36(2):ME20138. doi: 10.1264/jsme2.ME20138 (PMC8209445; doi:10.1264/jsme2.ME20138)
Supplement: Supplementary file 1 — Supplementary Material 1 [file 36_20138_s1.docx]

**Supplemental Material**

SI Figure 1 – class and genus level comparison of core- and pan-genomes; distribution of number of CLOGs with number of genomes at class and genus level. Core TS corresponds to the CLOGs found in all *Thermosynechococcus* genus members, and shared TS corresponds to CLOGs that are shared by at least 2 and at most 6 genus level genomes.

SI Table 1 – *Thermosynechococcus* genus core of CLOGs obtained from analysis at class level and their corresponding BLAST hit annotations when using the *T. vulcanus* protein sequences as query. The annotation is derived from the top hit (either the *T. vulcanus* annotation, or the multispecies annotation). The highlighted CLOGs are unique both at family and at class level comparisons.

SI Table 2 – Jinata and OHK specific CLOGs at class level with coverage and identity scores
